# Practice patterns of adjuvant radiotherapy in women with stage I to II endometrial carcinoma: a real-world multi-institutional analysis in China

**DOI:** 10.1186/s12905-023-02548-0

**Published:** 2023-08-09

**Authors:** Wenhui Wang, Tiejun Wang, Zi Liu, Jianli He, Xiaoge Sun, Wei Zhong, Fengjv Zhao, Xiaomei Li, Sha Li, Hong Zhu, Zhanshu Ma, Ke Hu, Fuquan Zhang, Xiaorong Hou, Lichun Wei, Lijuan Zou

**Affiliations:** 1 Department of Radiation Oncology, Peking Union Medical College Hospital, Chinese Academy of Medical Sciences and Peking Union Medical College, Beijing, China; 2 Department of Radiation Oncology, The Second Hospital Affiliated By Jilin University, Changchun, People’s Republic of China; 3 Department of Radiation Oncology, First Affiliated Hospital of Xi’an Jiaotong University, Xi’an, People’s Republic of China; 4 Department of Radiation Oncology, The General Hospital of Ningxia Medical University, Yinchuan, Ningxia People’s Republic of China; 5 Department of Radiation Oncology, The Affiliated Hospital of Inner Mongolia Medical University, Hohhot, Inner Mongolia People’s Republic of China; 6 Gynaecological Oncology Radiotherapy, The Affiliated Cancer Hospital of Xinjiang Medical University, Urumqi, People’s Republic of China; 7 Department of Radiation Oncology, Gansu Provincial Cancer Hospital, Lanzhou, Gansu People’s Republic of China; 8 Department of Radiation Oncology, Peking University First Hospital, Beijing, People’s Republic of China; 9 Department of Radiation Oncology, The 940Th Hospital of Joint Logistics Support Force of Chinesc People’s Liberation Army, Lanzhou, Gansu People’s Republic of China; 10 Department of Radiation Oncology, Xiangya Hospital Central South University, Changsha, Hunan People’s Republic of China; 11 Department of Radiation Oncology, Affiliated Hospital of Chi Feng University, Chifeng, Inner Mongolia People’s Republic of China; 12 Department of Radiation Oncology, Xijing Hospital, Air Force Medical University of PLA (the Fourth Military Medical University), Xi’an, People’s Republic of China; 13 Department of Radiation Oncology, The Second Hospital of Dalian Medical University, Dalian, People’s Republic of China

**Keywords:** External beam pelvic radiotherapy, Endometrial neoplasms, Vaginal brachytherapy, Practice patterns

## Abstract

**Background:**

This study aimed to report clinical practice patterns of postoperative radiotherapy for stage I to II endometrial carcinoma (EC) patients treated in 13 Chinese medical centers.

**Methods:**

We included early stage EC patients treated by hysterectomy and adjuvant RT between 2003 and 2017 from 13 institutions. Patients were classified into 4 risk groups based on ESMO-ESGO-ESTRO recommendations (2014).

**Results:**

A total of 1,227 cases were analyzed. Along the 15 years of the study, an increasing tendency was found towards administration for vaginal brachytherapy (VBT) alone, while the proportion of external beam pelvic radiotherapy (EBRT) alone remained stable in the corresponding period. When radiation modalities were stratified by risk groups, proportion of VBT alone significantly increased in all risk groups. The higher the risk, the later VBT became the main adjuvant treatment modality. However, EBRT alone or with VBT remained the main adjuvant method for high-risk patients.

There were 13 dose-fractionation schemes for VBT alone with the scheme of 30 Gy in 6 fractions prescribed at 0.5cm under the vaginal mucosa accounting for most. There were 17 schemes for VBT boost and the most common schedule was 10 Gy in 2 fractions. The upper 3–5cm part of vagina was the most frequent target. 89.6% of the practitioners performed two-dimensional VBT technique. The median dose for EBRT was 50 Gy. From 2003 to 2017, conventional radiotherapy was gradually replaced by three-dimensional conformal radiotherapy modality and intensity modulated radiotherapy.

**Conclusion:**

We report a significant shift from EBRT to VBT alone for high-intermediate-risk, intermediate-risk and low-risk EC patients from 2003 to 2017 while EBRT remained the main radiation modality for high-risk early stage patients. There has been remarkable heterogeneity among VBT dose fractionation schedules across China.

**Trial registration:**

The clinical trial ID was ChiCTR-PRC-17010712. It was authorized by the Institutional Review Board of Peking Union Medical College Hospital (N0. S-K139).

## Introduction

Tumor of the uterus is the secondly diagnosed gynecological tumor in China, with endometrial cancer (EC) accouting for most of it [1]. Due to the obvious clinical manifestations, majority of EC patients are diagnosed early. Surgery is the main procedure for EC confined to the uterus. For patients with risk factors, such as lympho-vascular space invasion, high grade and deep myometrial invasion, postoperative radiotherapy including external beam pelvic radiotherapy (EBRT) and/or vaginal brachytherapy (VBT) is recommended [2–4].

As many randomized researches have been performed [5–10] along with increasing institutional data, the clinical practice patterns of VBT and/or EBRT in the postoperative management of EC continues to change. In 2005, the American Brachytherapy Society (ABS) published data on clinical utilization of postoperative RT and in 2016, the data was updated [11, 12]. They demonstrated an increasing trend towards administration for VBT alone. Modh et al. [13] published data on trends in the real practice on utilization of VBT alone vs. EBRT or EBRT with VBT for stage I-II (FIGO 1988) EC by Surveillance, Epidemiology, and End Results database. He reported a significant increase for the use of VBT alone from 1995 to 2012, which was not limited to histological type, age, stage et al. Meanwhile, the proportion of EBRT with or without VBT decreased during the same period. However, there was limited data on clinical practice patterns of VBT and EBRT in China.

In this research, we would demonstrate radiotherapy pattern evolvement, treatment planning and dose fractionation schedules for early stage EC patients treated between 2003 to 2017 from 13 institutions in China.

## Methods and materials

Data of EC patients treated between 2003.1 and 2017.12 from 13 institutions in China was retrospectively reviewed. Patients of the following clinical characteristics were included: hysterectomy followed by postoperative RT, stage I to II, and complete clinical data. International Federation of Gynecology and Obstetrics (FIGO) (2009) staging system was used for all patients. Patients were stratified into high-risk (HR), high-intermediate-risk (HIR), intermediate-risk (IR) and low-risk (LR) groups based on ESMO-ESGO-ESTRO consensus (2014). This trial was retrospectively registered at 2017.02.23 and the clinical trial number is ChiCTR-PRC-17010712.

SPSS software was used for data analysis. The Kaplan–Meier method was performed to estimate survival outcomes. We considered a *p*-value < 0.05 statistically significant.

## Results

### Patients and treatments

One thousand two hundred twenty-seven early stage EC cases were included (Table 1). The pathology of most patients was endometrioid adenocarcinoma (92.7%, *n* = 1138). Percentage of HR, HIR, IR and LR groups were: 25.9% (*n* = 318), 19.2% (*n* = 235), 27.2% (*n* = 334) and 27.7% (*n* = 340), respectively.

**Table 1:** Baseline clinical characteristics for all patients treated from 2003 to 2017

	**Patients(*N* = 1,227)**	
**Clinical Characteristic**	No	%
**Age, years**		
Mean	56.2	
Range	23–86	
**Lymphadenectomy**		
No	361	29.4
Yes	866	70.6
Mean Number	23.7	
Range	1–99	
**Pathology type**		
Endometrioid Adenocarcinoma	1137	92.7
Nonendometrioid Carcinoma		
Mixed cell carcinoma	43	3.5
Serous carcinoma	22	1.8
Clear cell carcinoma	16	1.3
Undifferentiated carcinoma	6	0.5
Others	3	0.2
**Stage(FIGO 2009)**		
IA	566	46.1
IB	500	40.7
II	161	13.1
**Diameter**		
< 2 cm	141	11.5
≥ 2 cm	685	55.8
Missing	401	32.7
**Grade**^a^		
G1	395	34.7
G2	518	45.5
G3	219	19.2
Missing	6	0.5
**Myometrial invasion**		
< 1/2	638	52.0
≥ 1/2	581	47.4
Missing	8	0.7
**Invasion of lower uterine segment**		
No	885	72.1
Yes	342	27.9
**Involvement of cervix**		
No	975	79.5
Yes		
Cervical mucosa	91	7.4
Cervical stromal	161	13.1
**Lympho-vascular Space Invasion**		
Present	222	18.1
Absent	1005	81.9
**Chemotherapy**		
No	997	81.3
Yes	230	18.7

Abbreviation: *FIGO* International Federation of Gynecology and Obstetrics

^a^only for endometrioid adenocarcinoma

A hysterectomy and bilateral salpingo-oophorectomy surgery was performed for all cases. A total of 70.6% of all patients underwent lymphadenectomy. All patients underwent adjuvant RT, which included EBRT alone (*n* = 122), or with VBT (*n* = 491) and VBT alone (*n* = 614). EBRT was delivered to the pelvic lymphatic drainage regions and upper part of vagina. A total of 230 patients received chemotherapy. There were 154 patients in the HR group, 43 patients in the HIR group, 29 patients in the IR group, and 4 patients in the LR group, respectively. When used on the concurrent setting, cisplatin was administrated. When used as sequential chemotherapy, regimens such as paclitaxel, carboplatin/paclitaxel, cisplatin/doxorubicin and cisplatin/doxorubicin/paclitaxel were administrated. The number of adjuvant chemotherapy cycles ranged from 1 to 6.

### RT Pattern evolvement

**Fig. 1:**
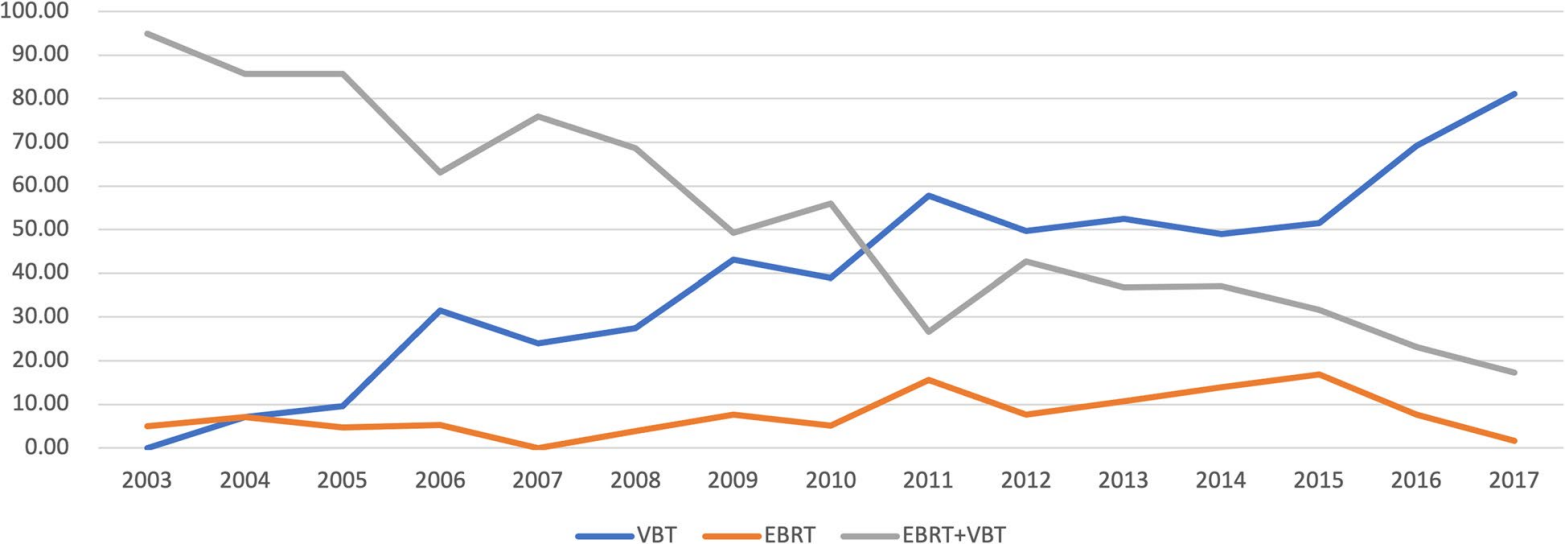
Radiotherapy pattern evolvement: percentage of different modalities from 2003 to 2017

An increasing tendency was found towards utilization for VBT alone overall. EBRT with VBT was the main treatment modality from 2003 to 2010 and VBT dominated from 2011. In 2003, there was no record of VBT alone, and the proportion rose to 81.1% in 2017. Meanwhile, the proportion of EBRT with VBT decreased. In 2003, 95.0% of patients received combined EBRT and VBT, and the proportion decreased to 17.3% in 2017. The proportion of EBRT alone remained stable in the corresponding period (Fig. 1).

In this research, trends of VBT alone, and EBRT with or without VBT were stratified by 4 risk groups (Table 2). Data demonstrated that the utilization of VBT alone increased remarkably among all groups from 2003 to 2017 while the proportion of EBRT with or without VBT decreased correspondingly in the period. In the LR group, VBT became the main adjuvant treatment early in the second five years (2008–2012). In the IR and HIR groups, VBT dominated in the third five years (2013–2017). In the HR group, EBRT with or without VBT remained the main adjuvant treatment modality from 2003 to 2017.

**Table 2:** Clinical practice of VBT alone and EBRT (with or without VBT) among different risk groups from 2003 to 2017

Patients(*N* = 1,227)					
		**2003–2007 (%)**	**2008–2012 (%)**	**2013–2017 (%)**	**p**
**Low-risk** (*N* = 340)	**VBT alone** (*N* = 243)	36.1 ^a^	61.7 ^a^	85.8 ^a^	**0.000**
	**EBRT with or without VBT** (*N* = 97)	63.9 ^a^	38.3 ^a^	14.2 ^a^	
**Intermediate -risk** (*N* = 334)	**VBT alone** (*N* = 184)	6.1 ^a^	48.6	67.0 ^a^	**0.000**
	**EBRT with or without VBT** (*N* = 150)	93.9 ^a^	51.4	33.0 ^a^	
**High-intermediate-risk** (*N* = 235)	**VBT alone** (*N* = 139)	0.0 ^a^	42.0	66.3 ^a^	**0.000**
	**EBRT with or without VBT** (*N* = 96)	100.0 ^a^	58.0	33.7 ^a^	
**High-risk** (*N* = 318)	**VBT alone** (*N* = 48)	0.0	13.0	17.4	**0.013**
	**EBRT with or without VBT** (*N* = 270)	100.0	87.0	82.6	

*Abbreviation: EBRT* External Beam Radiation, *VBT* Vaginal Brachytherapy

^a^adjusted residuals, only values greater than ± 3 were marked

### Treatment planning and dose fractionation

A total of 1105 patients received VBT, including 614 cases of VBT alone and 491 cases of VBT as a boost to EBRT. For patients receiving VBT, the vaginal irradiated target was mostly the proximal 3 to 5 cm (93.5%) of the vagina. The proximal 2 cm accounted for 6.3% and proximal 6 to 7 cm for 0.2% of the patients. The median irradiated vaginal length was 3 cm. Cylinders were the most commonly used applicators, followed by ovoids. A total of 1056 (95.6%) patients were treated with vaginal cylinders, including 154 (14.6%) single, central channel applicators and 902 (85.4%) multichannel vaginal applicators. For treatment of the vagina, the choice of applicator was both institution and doctor-dependent.

**Fig. 2:**
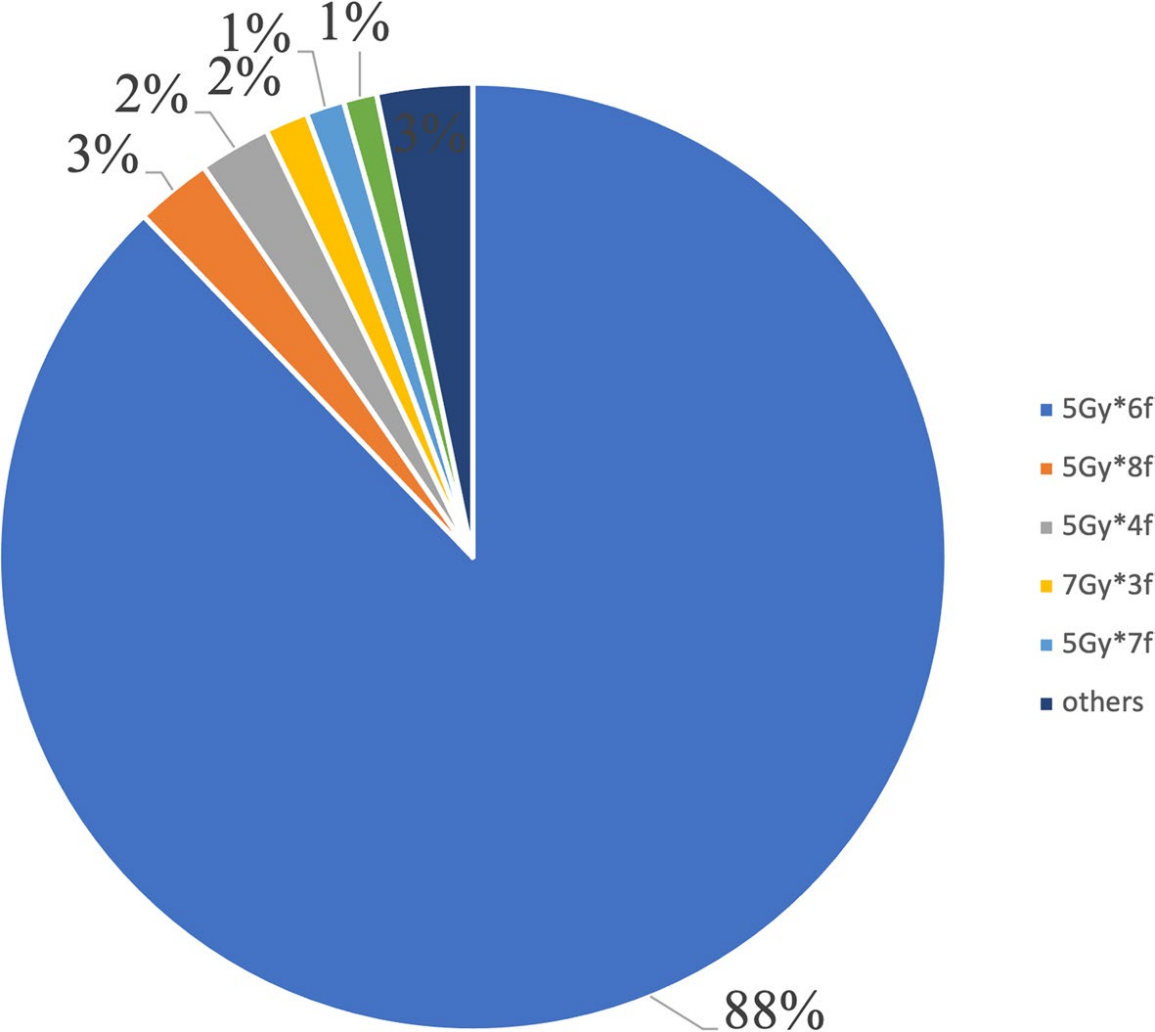
Dose-fractionation schemes for VBT alone

**Fig. 3:**
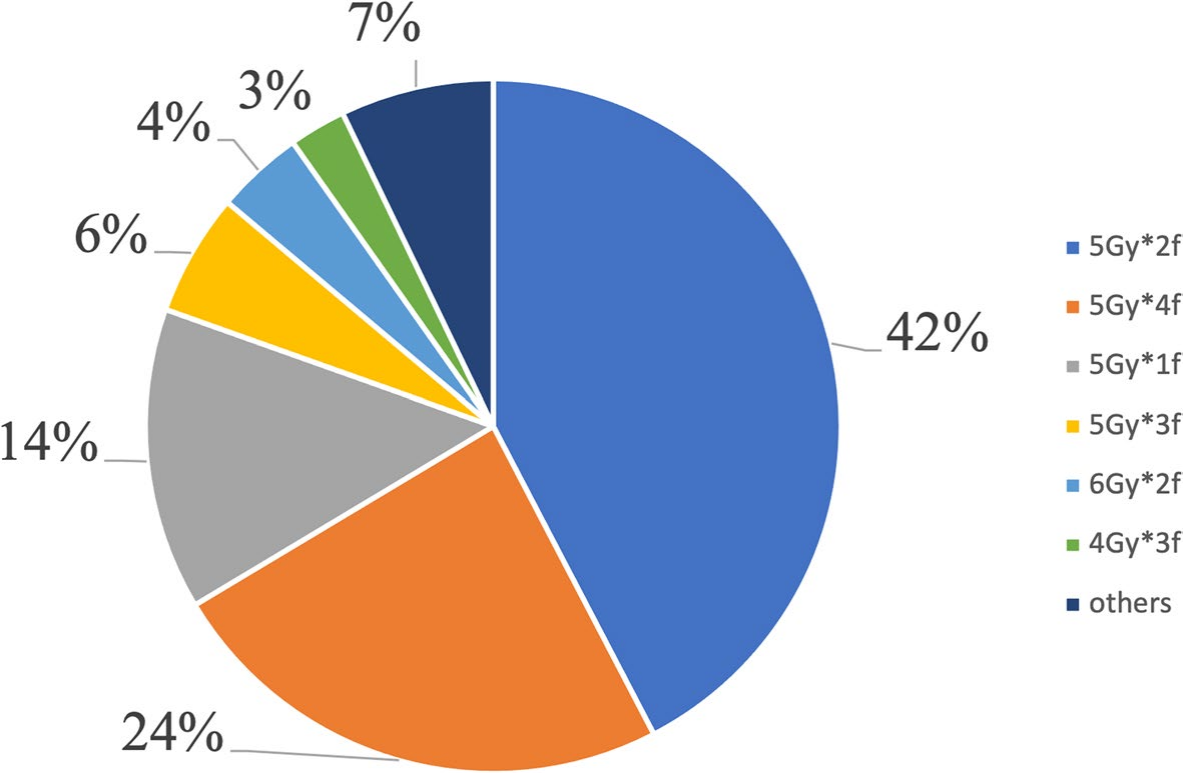
Dose-fractionation schemes for VBT boost

In terms of VBT planning pattern, 990 (89.6%) practitioners performed two-dimensional VBT technique, and others (115, 10.4%) used a three-dimensional VBT technique. For the former, all practitioners specified the dose to a 0.5 cm depth from the vaginal surface, while for the latter, clinical target volume was formed by expanding a 0.5 cm margin around the vaginal cylinder applicator excluding surrounding organs at risk. High-dose-rate appeared to be the only approach, accounting for 100% of all patients with diverse dose fractionation regimens. As to VBT alone, a total of 13 dose-fractionation schemes were performed, and for VBT boost, 17 dose-fractionation schemes were used. Overall, for VBT alone, the most common schedule was six fractions 5 Gy each, accounting for 87.8% (539/614) of all patients, followed by eight fractions 5 Gy each (2.6%, 16/614) (Fig. 2). For VBT boost, the most common schedule was two fractions 5 Gy each (42.4%, 208/491), followed by four fractions 5 Gy each (24.0%, 118/491) (Fig. 3).

In all, 613 patients received EBRT. The median dose and fractions for EBRT was 50 Gy (ranging from 39.6 to 54.0 Gy), and 25 fractions (ranging from 20 to 30 fx) respectively. For treatment planning, the computer tomography-based intensity modulated radiotherapy technique (IMRT) (*n* = 302, 49.3%) accounted for most, followed by conventional technique (*n* = 166, 27.0%) and three-dimensional conformal radiotherapy modality (3D-CRT) (*n* = 145, 23.7%). In 2003, all patients received conventional RT. Along the 15 years of the study, conventional RT has been gradually replaced by 3D-CRT and IMRT. 3D-CRT peaked in 2011 (73.7%) and then gradually replaced by IMRT.

As to timing of adjuvant radiotherapy, when VBT alone was used, most patients initiated radiotherapy at 4–6 (45.0%, *n* = 276) weeks postoperatively. 25.6% (*n* = 157), 20.8% (*n* = 128) and 8.6% (*n* = 53) of patients initiated VBT alone at 6–8, > 8 and 2–4 weeks postoperatively, respectively. When EBRT was used, timing of radiotherapy was equally distributed. Patients initiated EBRT at 4–6 (32.0%, *n* = 196), > 8 (25.3%, *n* = 155), 2–4 (24.0%, *n* = 147) and 6–8 (18.7%, *n* = 115) weeks, respectively. As to the median overall treatment time, it was 38 days (range, 26 to 83 days) for patients receiving EBRT with VBT and it was 13 days (range, 4 to 33 days) for patients receiving VBT alone.

### Survival

For all the patients, the overall follow-up time was 52 months (range, 2 to 204 months). The 5-year overall survival (OS), disease-free survival (DFS), local recurrence-free survival and distant metastasis-free survival rates were: 94.7%, 90.5%, 92.7% and 91.7%, respectively. When stratified by risk groups, 5-year OS rates were similar among HIR, IR and LR groups (95.9%, 95.5%, and 96.4%, respectively), while 5-year OS rate was lower for HR group (91.1%). The 5-year DFS rates were 84.8%, 90.2%, 92.2% and 94.1% for HR, HIR, IR and LR patients, respectively.

## Discussion

In this study, we reported the clinical practice patterns, treatment planning and dose prescription of different radiation modalities for early stage EC patients treated in 13 Chinese institutions. These results suggested a significant shift from EBRT to VBT for LR, IR and HIR stage I to II EC over the study period. Adjuvant EBRT with or without VBT remained the main radiation modality for HR stage I to II patients. There was great heterogeneity on dose-fractionation schedules which was slightly different from what was performed in the USA.

### RT Pattern evolvement

Radiotherapy patterns have evolved over the last two decades. From 2000 to 2010, prospective trials were conducted [5, 6, 8, 14] to compare postoperative EBRT with observation. The results demonstrated an increased local–regional control rate for patients receiving postoperative EBRT. With the promising data of the PORTEC-2 trial and other institutional researches [7, 9, 10], VBT alone was highly recommended for IR or HIR patients, as VBT was almost equally effective to EBRT in terms of disease control while women receiving VBT alone had a better life quality. The ABS performed a series of surveys on practice patterns of postoperative RT [11, 12] and reported an increasing trend towards referrals for VBT alone. Modh et al. [13] evaluated radiation patterns by Surveillance, Epidemiology, and End Results database for early stage patients treated from 1995 to 2012. He demonstrated the proportion of patients receiving VBT increased yearly while utilization of EBRT with VBT deceased significantly, which was consistent to our findings. However, a decreasing trend was shown on the use of EBRT alone from 1995 to 2012. That was different to our results that EBRT kept stable from 2003 to 2017. Besides, Modh et al. [13] reported that from 1995 to 2006, EBRT alone was the main adjuvant radiotherapy approach for all the EC patients and VBT dominated after 2007. This was different from our results that combined EBRT with VBT was the main treatment modality from 2003 to 2010. In the early years, some single-center experience on VBT as a boost have been reported [15–18]. Most data demonstrated EBRT with VBT and EBRT alone had equivalent local and regional control rates as well as OS [19, 20]. Therefore, combined EBRT with VBT was a choice in early years. The current view is, VBT as a boost to EBRT may be a choice for stage II (FIGO 2009) or grade 3 disease or lympho-vascular space invasion positive patients on an individual basis. As far as we know, most LR, IR and HIR patients in some centers in China received VBT alone since 2017. We look forward to our subsequent updated results.

As to trends in different risk groups, Modh et al. [13] demonstrated, the proportion of VBT alone increased significantly even for HR patients like stage II (FIGO 1988) disease or clear-cell/serous histology, which was consistent to our findings. However, in Modh’s research, VBT alone (67.4%) was the main RT modality for clear-cell/serous histology in 2012. In our research, EBRT with or without VBT was always preferred as the main RT modality in the HR group. Researches have been conducted for the proper treatment approach for high-risk early stage EC patients [21–24]. GOG249 [22] was designed to replace EBRT with combined chemotherapy and VBT. However, it failed to report a superiority of chemotherapy and VBT compared to EBRT on survival while the former had greater acute AE. In our study, the HR group included patients with stage IB grade 3 disease, stage II disease and serous/ clear-cell histology. Due to the small amount of HR patients, analysis stratified by various factors was not carried out which might interfere comparation to Modh’s research.

### Treatment planning and dose fractionation

There was significant variety among VBT dose-fractionation schemes, as there were 13 different schedules as monotherapy and 17 as a boost, not to mention the diversity of dose-fractionation schemes of combined EBRT with VBT.

When VBT alone was used as adjuvant monotherapy, as Harkenrider et al. [2] summarized, in the US, the most commonly performed regimen was 3 fractions 7 Gy each prescribed to a 0.5-cm depth, followed by 5 fractions 6 Gy each prescribed to the vaginal surface. Since different doses per fraction would result in different biological effects, we used EQD_2_10 (Equivalent Dose in 2 fractions, α/β = 10) to compare different dose-fractionation effects. The total 5 mm depth EQD_2_10 (Gy) for 3 fractions 7 Gy each was 29.75 Gy and for 5 fractions 6 Gy each was 22.59. Different from that in the USA, the most commonly used scheme of VBT alone in China was 30 Gy in 6 fractions prescribed to 5 mm below the vaginal mucosa (total 5-mm depth EQD_2_10 (Gy): 37.5 Gy), followed by 8 fractions 5 Gy each prescribed to 5 mm below the vaginal mucosa (total 5-mm depth EQD_2_10 (Gy): 50 Gy). Therefore, the prescription dose of VBT alone in China has been higher than that in the United States. However, vaginal recurrence rates after surgery and adjuvant VBT were low in stage I to II EC patients and were not significantly different by VBT dose fractionation schedules [25].

When VBT was used as an EBRT boost, the dose fractionation schedules were variable, as there were diverse EBRT and VBT dose prescriptions. In this research, the median dose for EBRT was 50 Gy. Meanwhile, the commonly used scheme for VBT boost was 2 fractions 5 Gy each followed by 4 fractions 5 Gy each prescribed to a 0.5 cm depth. The recommended fractionation schedules by ABS included 45 Gy EBRT + 15–18 Gy in 3 fractions VBT to the vaginal surface or 50.4 Gy EBRT + 12 Gy in 2 fractions VBT to the vaginal surface [26]. The dose prescriptions were slightly different from those in the US.

The technical details and prescription doses for the administration of postoperative RT have been described in different guidelines. There were various dose fractionation prescriptions practically performed. There was no common view on the superiority of one scheme over others. Therefore, the ABS did not propose the best regimen [2, 26]. The prescription dose was the result of the balance between efficacy and toxicity, and was adjusted by different radiation oncologists in different regions. We do not aim to seek for the best treatment options but rather simply describe how we are actually performing evidenced-based care.

### Limitations

The following were the limitations of this research. As a retrospective research, there might be selection bias and some pathology and chemotherapy details were not complete. As a multicenter study, the number of centers included in this research was too small to summarize the current situation of EC treatment in China. Besides, in-depth group analysis could not be carried out due to the sample size. Despite these limitations, this was the first and largest sample sized study that has investigated the real practice patterns of adjuvant VBT and EBRT with or without VBT from 2003 to 2017 for early stage patients in China.

## Conclusions

The utilization of postoperative VBT alone has increased remarkably in all risk groups and EBRT with or without VBT remained stable as the main treatment modality in the HR groups along the 15 years of the study. There has been significant heterogeneity among VBT dose fractionation schemes which was different from that in the US.

## Data Availability

The datasets used and analyzed during the current research are available from the corresponding author upon reasonable request.

## Abbreviations

EC: Endometrial cancer
EBRT: External beam radiation
VBT: Vaginal brachytherapy
RT: Radiotherapy
ESMO: European Society for Medical Oncology
ESTRO: European Society for Radiotherapy & Oncology
ESGO: European Society of Gynaecological Oncology
FIGO: International Federation of Gynecology and Obstetrics
IMRT: Intensity modulated radiation therapy
3D-CRT: Three-dimensional conformal radiotherapy
OS: Overall survival
DFS: Disease-free survival

## Declarations

### Trial registration number

The trial was retrospectively registered at 2017.02.23 and the registration number is ChiCTR-PRC-17010712.
